# Diffusion weighted and dynamic contrast enhanced MRI as an imaging biomarker for stereotactic ablative body radiotherapy (SABR) of primary renal cell carcinoma

**DOI:** 10.1371/journal.pone.0202387

**Published:** 2018-08-16

**Authors:** Hayley M. Reynolds, Bimal K. Parameswaran, Mary E. Finnegan, Diana Roettger, Eddie Lau, Tomas Kron, Mark Shaw, Sarat Chander, Shankar Siva

**Affiliations:** 1 Department of Physical Sciences, Peter MacCallum Cancer Centre, Melbourne, Victoria, Australia; 2 Sir Peter MacCallum Department of Oncology, The University of Melbourne, Melbourne, Victoria, Australia; 3 Cancer Imaging, Peter MacCallum Cancer Centre, Melbourne, Victoria, Australia; 4 Department of Imaging, Imperial College Healthcare NHS Trust, London, United Kingdom; 5 Department of Bioengineering, Imperial College London, London, United Kingdom; 6 Image Analysis Group, London, United Kingdom; 7 Division of Radiation Oncology, Peter MacCallum Cancer Centre, Melbourne, Victoria, Australia; King's College London, UNITED KINGDOM

## Abstract

**Purpose:**

To explore the utility of diffusion and perfusion changes in primary renal cell carcinoma (RCC) after stereotactic ablative body radiotherapy (SABR) as an early biomarker of treatment response, using diffusion weighted (DWI) and dynamic contrast enhanced (DCE) MRI.

**Methods:**

Patients enrolled in a prospective pilot clinical trial received SABR for primary RCC, and had DWI and DCE MRI scheduled at baseline, 14 days and 70 days after SABR. Tumours <5cm diameter received a single fraction of 26 Gy and larger tumours received three fractions of 14 Gy. Apparent diffusion coefficient (ADC) maps were computed from DWI data and parametric and pharmacokinetic maps were fitted to the DCE data. Tumour volumes were contoured and statistics extracted. Spearman’s rank correlation coefficients were computed between MRI parameter changes versus the percentage tumour volume change from CT at 6, 12 and 24 months and the last follow-up relative to baseline CT.

**Results:**

Twelve patients were eligible for DWI analysis, and a subset of ten patients for DCE MRI analysis. DCE MRI from the second follow-up MRI scan showed correlations between the change in percentage voxels with washout contrast enhancement behaviour and the change in tumour volume (ρ = 0.84, p = 0.004 at 12 month CT, ρ = 0.81, p = 0.02 at 24 month CT, and ρ = 0.89, p = 0.001 at last follow-up CT). The change in mean initial rate of enhancement and mean Ktrans at the second follow-up MRI scan were positively correlated with percent tumour volume change at the 12 month CT onwards (ρ = 0.65, p = 0.05 and ρ = 0.66, p = 0.04 at 12 month CT respectively). Changes in ADC kurtosis from histogram analysis at the first follow-up MRI scan also showed positive correlations with the percentage tumour volume change (ρ = 0.66, p = 0.02 at 12 month CT, ρ = 0.69, p = 0.02 at last follow-up CT), but these results are possibly confounded by inflammation.

**Conclusion:**

DWI and DCE MRI parameters show potential as early response biomarkers after SABR for primary RCC. Further prospective validation using larger patient cohorts is warranted.

## Trial registration

ANZCTR, U1111-1132-5574 https://clinicaltrials.gov/show/NCT01676428

## Introduction

Stereotactic ablative body radiotherapy (SABR) is a novel high-precision treatment modality suitable for patients with primary renal cell carcinoma (RCC) [1]. Recently, our group completed a prospective pilot clinical trial in this context (FASTRACK, trial number U1111-1132-5574) [2,3]. Dose delivered was dependent on tumour size with lesions ≤5 cm diameter receiving a single fraction of 26 Gy and larger lesions three fractions of 14 Gy prescribed to the 99% of the target volume. Subsequent studies using SPECT/CT imaging and ^51^Cr-EDTA measurements showed the change in renal function after SABR in these patients was correlated with the dose delivered [4,5].

It is known that SABR of RCC can achieve high rates of local control, however, ablative doses of radiotherapy can lead to cell-kill and cancer control without necessarily resulting in tumour morphological change, and residual non-viable tumour architecture can remain for sustained periods post-treatment [6]. Despite this, anatomical size-based CT assessment such as the Response Evaluation Criteria in Solid Tumors (RECIST) criteria [7] are often used to assess response to therapy.

MRI offers an alternate means to evaluate tumour response [8] and is currently rated as equivalent to CT by the American College of Radiology for RCC post-treatment follow-up [9]. Multi-parametric MRI offers important advantages over CT for assessing the biological effect of radiotherapy, most notably by assessing tissue perfusion using dynamic contrast enhanced MRI [10–12] and for characterising diffusion using diffusion weighted imaging (DWI) [13]. Studies into DWI and DCE MRI for RCC have been increasing in recent years as described in a number of review articles [14–16], particularly for their potential as treatment response biomarkers to targeted therapies indicating the presence of radiation-induced oedema, changes in cellularity, changes in vascularity and necrosis. Currently, however, no MRI-derived biomarkers are used in routine clinical practice for primary or metastatic RCC and no studies have been performed to investigate the use of mpMRI for assessing response to SABR treatment of RCC.

In this study we explored the utility of diffusion and perfusion changes shown in primary RCC tumours after SABR as an early biomarker of treatment response using DWI and DCE MRI.

## Methods

### Patients

**Fig 1:**
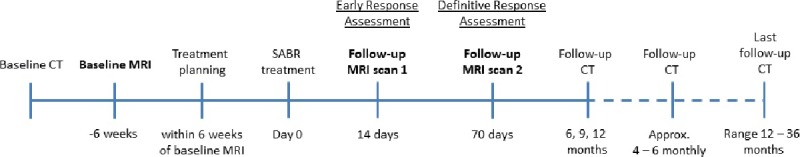
Schema showing the time points MRI and CT images were acquired relative to when SABR treatment was given (Day 0).

This was a single institutional prospective clinical trial with ethics review board approval at the Peter MacCallum Cancer Centre. Patients with primary RCC who were not operative candidates received SABR and underwent MRI scanning with Gadolinium contrast enhancement unless having medical contraindications (for example, inadequate renal function or a cardiac pacemaker). A schematic of scheduled MRI and CT imaging time points and SABR treatment delivery time is shown in Fig 1, with further details given below.

### Radiotherapy treatment

Radiotherapy fractionation was determined based on tumour size. For primary RCC with a tumour size of ≤5 cm diameter, a single fraction of 26 Gy was prescribed, and for larger tumours three fractions of 14 Gy was prescribed to the 99% of the target volume. The clinical target volume (CTV) was defined using an internal target volume with no margin for microscopic disease, as defined by the tumour delineated from 10 respiratory sorted bins on a 4D CT. The planning target volume (PTV) was defined by applying a 5 mm isotropic expansion to the CTV. Three-fraction treatment was delivered on non-consecutive days over one week. Treatment was delivered using a 3D conformal technique on a conventional linear accelerator system as previously described [2]. Peak dose within the PTV was typically 125%. Immobilisation of patients was achieved using a dual-vacuum immobilisation technique using a commercially available platform [17].

### CT imaging

Tumour measurements were obtained on CT before SABR treatment (baseline), and three monthly for the first year at 6, 9 and 12 months. After this, CT scans were taken at the treating clinician’s discretion, ranging from 4 to 6-monthly until the trial was closed. No other tumour directed therapy was delivered in the interval. In this study, we analysed the scheduled 6 month, 12 month, 24 month and the last follow-up CT, where the last follow-up CT ranged between 12 months to 36 months after SABR treatment (see S1 Table for further details). To compute tumour volume in mm^3^ from each CT scan we assumed the tumour was an ellipsoid shape according to the following equation [18]:

$$\frac{\pi }{6}\times AP\times TR\times CC$$ (1)

where AP is the measurement in the anterior-posterior direction, TR is the measurement in the transverse direction, and CC is the measurement in the cranio-caudal direction (all in millimetres). We then computed the change in tumour volume at each CT scan by subtracting the tumour volume at baseline CT.

### MRI data acquisition

MRI scans including DWI and DCE MRI sequences were obtained for each eligible patient, prior to treatment (baseline) and at two follow up time points scheduled for 14 days and 70 days after SABR (Fig 1). Images were acquired using a body coil and spine coil on a 3T Siemens Trio Tim scanner (Siemens Medical Solutions, Erlangen, Germany).

Diffusion weighted images (DWI) were collected using an axial echo-planar imaging sequence with b values of 50, 400 and 800 s/mm^2^, acquisition matrix = 128 x 128, FOV = ranging from 360 x 360 mm to 420 x 420 mm, with 18–24 transverse slices with thickness = 4mm and spacing between slices = 6 mm. ADC maps were generated using a mono-exponential fit to the DWI data in Siemens software.

DCE MRI data was obtained using a 3D T1-weighted TWIST sequence and injecting each patient with a 10ml bolus injection of contrast agent Dotarem (gadoterate meglumine, Guerbet, USA), followed by a saline flush. The dynamic scans were acquired in the axial plane with acquisition matrix = 192 x 192, FOV = 400 x 400 mm, flip angle = 20° with 20 transverse partitions at 3.6 mm slice thickness and repeated 30 times at 5.2 second intervals. This time period was the same for all scans except the pre-treatment scan for patient 2 who had images repeated 50 times at the same interval of 5.2 seconds. The dynamic scan was preceded by a pre-contrast TWIST sequence with variable flip angles (5°, 10°, 15°, 20°, 30°) to enable calculation of a T1 map, however this data was not utilised (see below).

### DWI analysis

Changes in water diffusivity within each tumour after SABR was assessed by analysing the ADC maps computed from DWI data. ADC maps were read into 3D Slicer software [19] and the tumour volume contoured directly onto ADC map image slices by an experienced radiologist (BP) using visual correlation to tumour margins seen clearly on the T2-weighted and post contrast MR images. The ADC values in all tumour voxels were then extracted and mean, kurtosis and skewness measures were calculated using MATLAB software (MATLAB Release 2015b, The MathWorks, Inc., Natlick, Massachusetts, USA).

### DCE semi-quantitative parametric maps

Dynamika software (Image Analysis Group, London, UK) [20] was used to investigate tumour perfusion. DCE images were 2D motion corrected to the first post-contrast image showing clear enhancement, after which semi-quantitative parametric maps were created by fitting a continuous piecewise linear function to the signal intensity curve of each voxel (as shown in Figure A in S1 Fig). This involved fitting four separate models (defining baseline, uptake, plateau and washout phases) to the voxel signal intensity curves using least-squares fitting to give models with the smallest error. The first two images were set as the baseline signal. Semi-quantitative parametric maps were then produced by extracting parameters from the models, including the time of onset of the contrast agent (T_onset_), the initial rate of enhancement (IRE), the maximum enhancement (ME), time of washout of the contrast agent (T_washout_), the initial rate of washout (IRW). Additionally, each voxel’s contrast agent uptake curve was classified into one of four behaviours to give a Gadolinium (GD) map. The uptake curve behaviours included: (1) persistent enhancement where a voxel had increased in intensity however a maximum enhancement had not been reached, (2) plateau enhancement where there was both an increase in signal intensity and a plateau phase, (3) washout enhancement where an increase, plateau and a decrease in signal intensity was observed and (4) no enhancement where there was negligible change to the signal in a voxel over time (see Figure B in S1 Fig). Voxels with no enhancement were subsequently excluded from the calculation of mean semi-quantitative parametric and pharmacokinetic values.

### DCE pharmacokinetic maps

Pharmacokinetic models were fitted to the DCE MRI data using Dynamika software (Image Analysis, London, UK) [20] in order to assess the Ktrans parameter, which is the volume transfer constant between blood plasma and the extra-vascular extra-cellular space. The extended Tofts model [21] was implemented due to the inclusion of an additional term in the model, which accounts for blood vessels in the tissue contributing to the overall signal which is more appropriate for highly vascularised kidneys. To fit the extended Tofts model the frame number just prior to when signal increase was observed in the tumour (Tonset frame) was specified, and the arterial input function (AIF) was defined manually in the aorta. Relaxivity of the contrast agent was 2.8 Lmmol^-1^s^-1^ [22], and the T1 value for the arterial blood relaxation time was set to 1664 ms [23]. The motion in some patients’ pre contrast variable flip angle scans was shown to impact the accuracy of tissue T1 calculation and therefore a fixed T1 value for all patients was used to provide a more consistent approach. The T1 relaxation time for the kidney was defined as 1142 ms, measured previously in cortical kidney tissue at 3T [24]. In addition, iAUGC60 which is the initial area under the contrast agent concentration curve for the first 60 second post-injection, was computed.

Tumour volumes were contoured by an experienced radiologist (BP) on the reference 3D TWIST images used for 2D motion correction, using visual correlation to the tumour margins as shown on the T2-weighted and post contrast MR images. Mean perfusion statistics were extracted from all semi-quantitative parametric and pharmacokinetic maps. Histogram analysis has shown limited utility for DCE data of primary RCC [25], so for this study only mean DCE MRI values were evaluated.

### Statistics

The changes in mean ADC values and mean DCE parameters were calculated for each patient’s post treatment MR images relative to baseline MRI. Spearman rank correlation coefficients were calculated in R statistical software [26] to compare the change in each DWI and DCE measurement versus the percentage change in tumour volume between each follow-up CT and baseline CT. P-values less than or equal to 0.05 were considered statistically significant.

## Results

### Study population

Characteristics for the twelve patients eligible for analysis are detailed in Table 1. Age ranged from 72–89 years, there were 8 male patients and 4 female patients, where 7 patients had a tumour in the right kidney and 5 patients had a tumour in the left kidney. All had clear cell RCC histology except for one who had a papillary RCC tumour (patient 5). Four patients were classified as partial responders (PR) based on the RECIST 1.1 criteria, seven patients had stable disease (SD) and one patient had progressive disease (PD). There were two patients with missing CT data at 6 months, and two patients could not be assessed at 24 months since their last follow-up CT had been performed close to the trial closing date at 12 months and 18 months respectively. S1 Table further details the number of days between each MRI and CT scan relative to the start of SABR treatment.

**Table 1:** Patient details.

Patient #	Age	Sex	Kidney	Histology	Treatment schedule	Baseline measures			CT tumour size (AP x TR x CC) [mm]					Follow-up duration (months)	RECIST 1.1 response*
						eGFR (ml/min)	Creatinine (umol/L)	CCS	Baseline CT	6 month CT	12 month CT	24 month CT	Last follow-up CT		
1	87	M	Left	Clear Cell	42 Gy/3Fx	62	104	9	43x49x72	51x60x60	51x35x48	24x22x29	24x22x29	26	PR
2	73	M	Left	Clear Cell	26 Gy/1Fx	70	87	8	31x38x37	27x34x31	30x26x29	20x16x21	17x17x18	36	PR
3	86	M	Right	Clear Cell	42 Gy/3Fx	43	144	12	38x61x56	-	27x43x44	28x39x39	28x39x39	24	PR
4	76	M	Right	Clear Cell	26 Gy/1Fx	61	108	8	30x21x41	28x20x33	26x16x26	24x14x26	24x14x26	24	PR
5	78	M	Left	Papillary	26 Gy/1Fx	58	113	9	41x30x37	-	29x21x30	26x16x35	39x19x30	30	SD
6	78	F	Right	Clear Cell	26 Gy/1Fx	>70	72	6	30x34x34	32x37x42	27x33x30	27x29x30	26x30x29	26	SD
7	78	F	Right	Clear Cell	42 Gy/3Fx	45	109	9	64x67x67	61x70x76	58x66x77	63x67x76	64x68x75	26	SD
8	78	M	Left	Clear Cell	26 Gy/1Fx	39	159	11	19x28x36	29x25x33	22x28x31	24x32x31	24x32x31	24	SD
9	89	F	Left	Clear Cell	42 Gy/3Fx	47	102	10	53x45x53	57x47x53	57x51x57	-	58x48x59	18	SD
10	72	M	Right	Clear Cell	26 Gy/1Fx	64	105	9	29x35x31	35x44x39	31x35x40	-	31x35x40	12	SD
11	77	M	Right	Clear Cell	42 Gy/3Fx	41	154	6	34x58x56	37x61x63	36x60x64	42x61x61	42x61x61	24	SD
12	72	F	Right	Clear Cell	26 Gy/1Fx	70	71	10	23x22x21	24x19x24	23x21x23	29x27x25	28x28x26	33	PD

eGFR = estimated Glomerular Filtration Rate, CCS = Charlson Comorbidity Score, AP = anterior-posterior, TR = transverse, CC = cranio-caudal, PR = partial response, SD = stable disease, PD = progressive disease.

*RECIST 1.1 response given based on the last follow-up CT tumour measurements.

**Fig 2:**
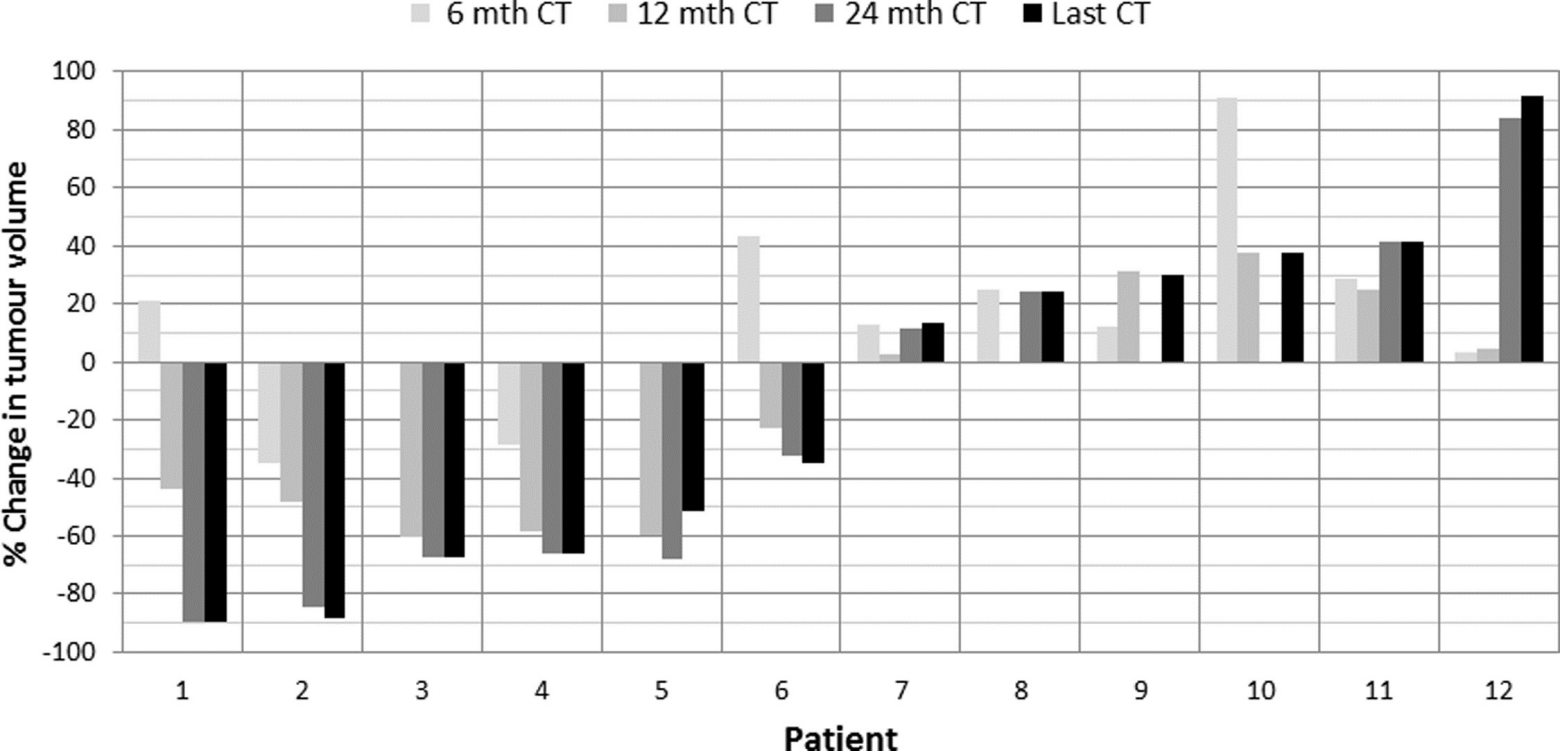
Percentage change in tumour volume between the 6 month, 12 month, 24 month and last follow-up CT relative to baseline CT.

Fig 2 displays the percentage change in tumour volume at the 6 month, 12 month, 24 month and the last follow-up CT relative to baseline CT for each patient. At the last follow-up CT the change in tumour volume ranged from -89.9 to 91.8%. All four partial responders and two patients with stable disease (patients 1–6) showed a decrease in percentage tumour volume. All remaining patients showed a tumour volume increase after treatment with the maximum tumour growth of 91.8% in patient 12.

### DWI analysis

**Fig 3:**
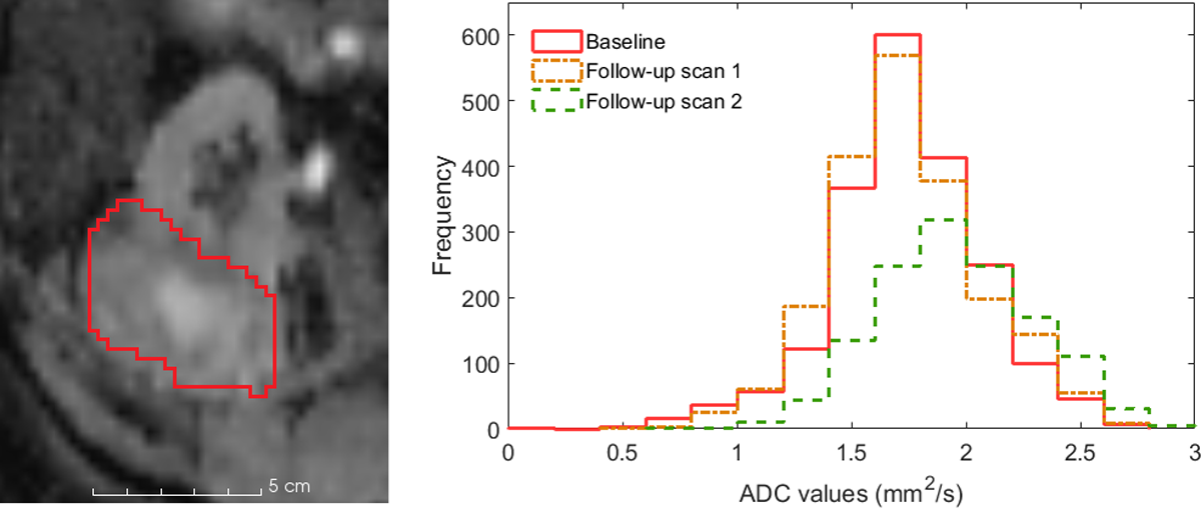
Baseline ADC map with tumour contour in red for patient 3 and associated histogram showing the frequency of ADC values in each MRI scan.

Fig 3 shows an example ADC map for patient 3 with tumour contours drawn. Spearman rank coefficients calculated between the change in each ADC statistic after SABR relative to baseline MRI and the change in tumour volume from CT are given in Table 2. There were 10 patients with complete data for DWI analysis at the 6 month CT, all 12 patients for the 12 month CT and last follow-up CT analysis, and 10 patients for the 24 month CT analysis.

An increase in mean and median ADC after SABR treatment was observed in eight patients at the second follow-up MRI scan relative to baseline while seven of these also had an increase in mean and median ADC at the first follow-up MRI scan relative to baseline (see Figure A in S2 Fig).

**Table 2:** Correlation coefficients between the change in ADC measures relative to baseline MRI and the change in tumour volume from CT. P-values ≤ 0.05 are in bold.

	Follow-up MRI scan 1				Follow-up MRI scan 2			
	6 month CT (n = 10)	12 month CT (n = 12)	24 month CT (n = 10)	Last CT (n = 12)	6 month CT (n = 10)	12 month CT (n = 12)	24 month CT (n = 10)	Last CT (n = 12)
Mean	0.02 (0.97)	-0.19 (0.56)	0.59 (0.08)	0.22 (0.50)	-0.22 (0.54)	-0.55 (0.07)	-0.13 (0.73)	-0.22 (0.50)
Median	0.12 (0.76)	-0.11 (0.73)	**0.65 (0.05)**	0.31 (0.32)	-0.25 (0.49)	-0.55 (0.07)	-0.14 (0.71)	-0.22 (0.48)
Kurtosis	-0.25 (0.49)	**0.66 (0.02)**	0.56 (0.10)	**0.69 (0.02)**	-0.33 (0.35)	0.41 (0.18)	0.24 (0.51)	0.28 (0.38)
Skewness	0.15 (0.68)	-0.15 (0.65)	**-0.71 (0.03)**	-0.55 (0.07)	0.41 (0.25)	0.40 (0.20)	-0.02 (0.97)	0.16 (0.62)

As indicated by correlation coefficients in Table 2, despite there being a significant correlation between change in mean ADC in the first follow-up MRI scan with the change in tumour volume at 12 month CT, there was no clear and consistent pattern in the change in mean or median ADC to distinguish response using DWI. Those with increased mean and median diffusion at the second follow-up MRI scan included three partial responders according to RECIST criteria, four with stable disease and one with progressive disease; while the three patients with decreased diffusion included a partial responder (patient 1) and three patients with stable disease (patients 9–11).

A positive correlation was shown between the change in ADC kurtosis and the change in tumour volume at the first follow-up MRI scan from the 12 month CT scan onwards which was considered significant at the 12 month and last follow-up CT (ρ = 0.66, p = 0.02 and ρ = 0.69, p = 0.02 respectively). In addition, the change in ADC skewness at the first follow-up MRI scan was negatively correlated with the percentage change in tumour volume from the 12 month CT scan onwards, but was only significant at the 24 month CT time point (ρ = -0.71, p = 0.03). At the second follow-up MRI scan the change in ADC kurtosis showed a positive correlation from the 12 month CT onwards however this correlation and all other ADC parameter correlations were considered statistically not significant. The raw ADC kurtosis values for each MRI scan can be seen in Figure B in S2 Fig.

### DCE analysis

**Fig 4:**
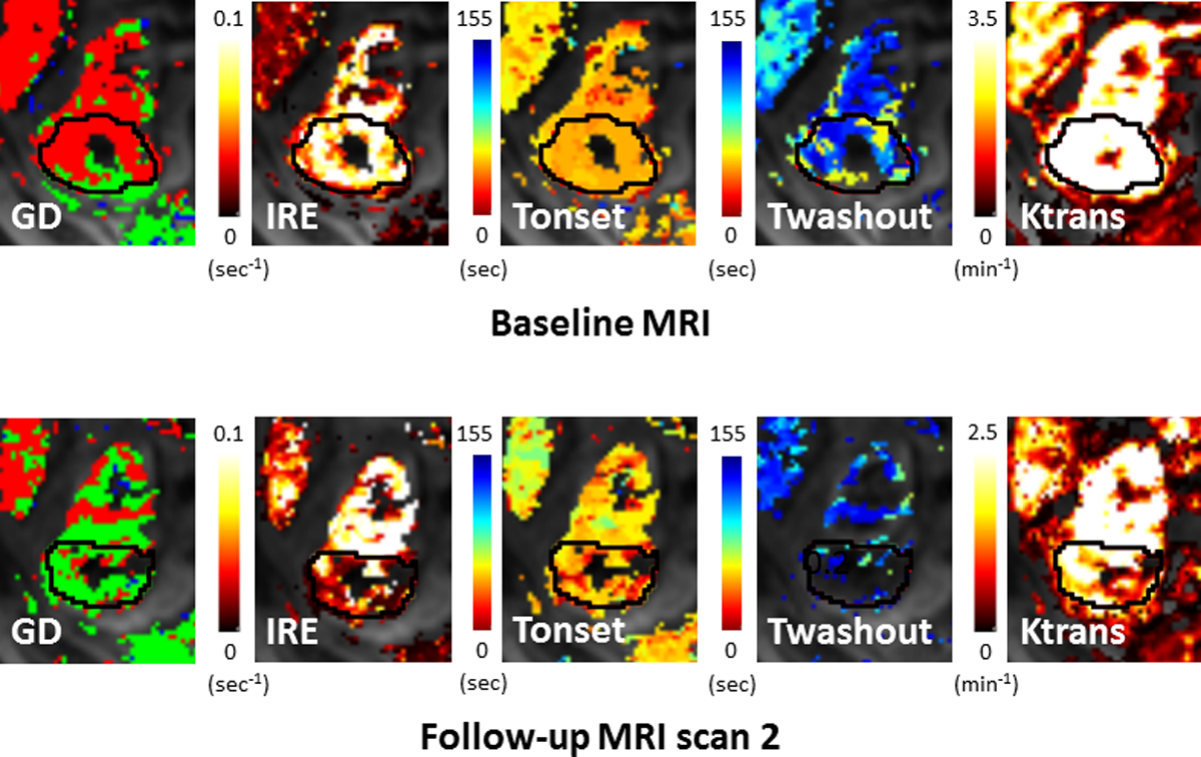
Selected DCE maps at baseline and follow-up MRI scan 2 for patient 3, left to right: The contrast enhancement GD map (green = plateau, red = washout, blue = persistent), IRE, Tonset, Twashout and Ktrans. The corresponding image between MRI scans has been visually selected.

Table 3 details the correlation coefficients between the change in mean DCE parameter with the change in tumour volume shown on CT. Patient 5 was excluded in this analysis due to its papillary RCC histology which is known to show very different enhancement behaviour to clear cell RCC [27], and patient 8 was excluded due to noisy data which meant that no tumour voxels in their second follow-up MRI scan fit adequately to the parametric models. Hence, there were 9 patients with complete data for DCE MRI analysis at the 6 month CT, 10 patients for the 12 month CT and last follow-up CT analysis, and 8 patients for the 24 month CT analysis. Fig 4 shows example semi-quantitative and pharmacokinetic parametric maps for patient 3.

**Table 3:** Correlation coefficients between the change in mean DCE semi-quantitative parametric and pharmacokinetic values relative to baseline MRI and the change in tumour volume from CT. P-values ≤ 0.05 are in bold.

	Follow-up MRI scan 1				Follow-up MRI scan 2			
	6 month CT (n = 9)	12 month CT (n = 10)	24 month CT (n = 8)	Last CT (n = 10)	6 month CT (n = 9)	12 month CT (n = 10)	24 month CT (n = 8)	Last CT (n = 10)
Mean ME	0.60 (0.10)	-0.10 (0.79)	-0.24 (0.58)	-0.31 (0.39)	0.03 (0.95)	0.03 (0.95)	-0.24 (0.58)	-0.18 (0.63)
Mean IRE	-0.56 (0.12)	-0.12 (0.75)	-0.25 (0.55)	-0.32 (0.37)	-0.15 (0.71)	**0.65 (0.05)**	0.71 (0.06)	**0.78 (0.01)**
Mean IRW	0.10 (0.81)	-0.08 (0.84)	-0.24 (0.58)	-0.30 (0.41)	0.25 (0.51)	0.06 (0.87)	0.08 (0.84)	0.00 (1.00)
Mean Tonset	-0.23 (0.55)	-0.36 (0.31)	-0.52 (0.20)	-0.38 (0.28)	0.47 (0.21)	0.07 (0.86)	0.57 (0.15)	0.32 (0.37)
Mean Twashout	0.45 (0.23)	0.15 (0.68)	0.64 (0.10)	0.56 (0.10)	-0.23 (0.55)	-0.45 (0.19)	0.19 (0.66)	-0.02 (0.97)
% Washout voxels	0.20 (0.61)	0.50 (0.14)	0.62 (0.11)	0.60 (0.07)	0.33 (0.39)	**0.84 (0.004)**	**0.81 (0.02)**	**0.89 (0.001)**
% Plateau voxels	-0.15 (0.71)	-0.53 (0.12)	-0.62 (0.11)	**-0.75 (0.02)**	0.03 (0.95)	-0.27 (0.45)	-0.31 (0.46)	-0.28 (0.43)
% Persistent voxels	-0.10 (0.81)	-0.21 (0.56)	-0.55 (0.17)	-0.39 (0.26)	0.08 (0.84)	-0.38 (0.28)	-0.38 (0.36)	-0.43 (0.22)
% Non-enhancing voxels	-0.18 (0.64)	-0.13 (0.73)	0.48 (0.24)	0.39 (0.26)	-0.22 (0.58)	-0.35 (0.33)	-0.60 (0.13)	-0.55 (0.10)
Mean Ktrans	-0.22 (0.58)	0.30 (0.41)	0.21 (0.62)	0.18 (0.63)	0.17 (0.68)	**0.66 (0.04)**	0.40 (0.33)	0.56 (0.10)
Mean iAUGC60	-0.12 (0.78)	-0.21 (0.56)	-0.43 (0.30)	-0.44 (0.20)	-0.43 (0.25)	0.20 (0.58)	0.26 (0.54)	0.26 (0.47)

**Fig 5:**
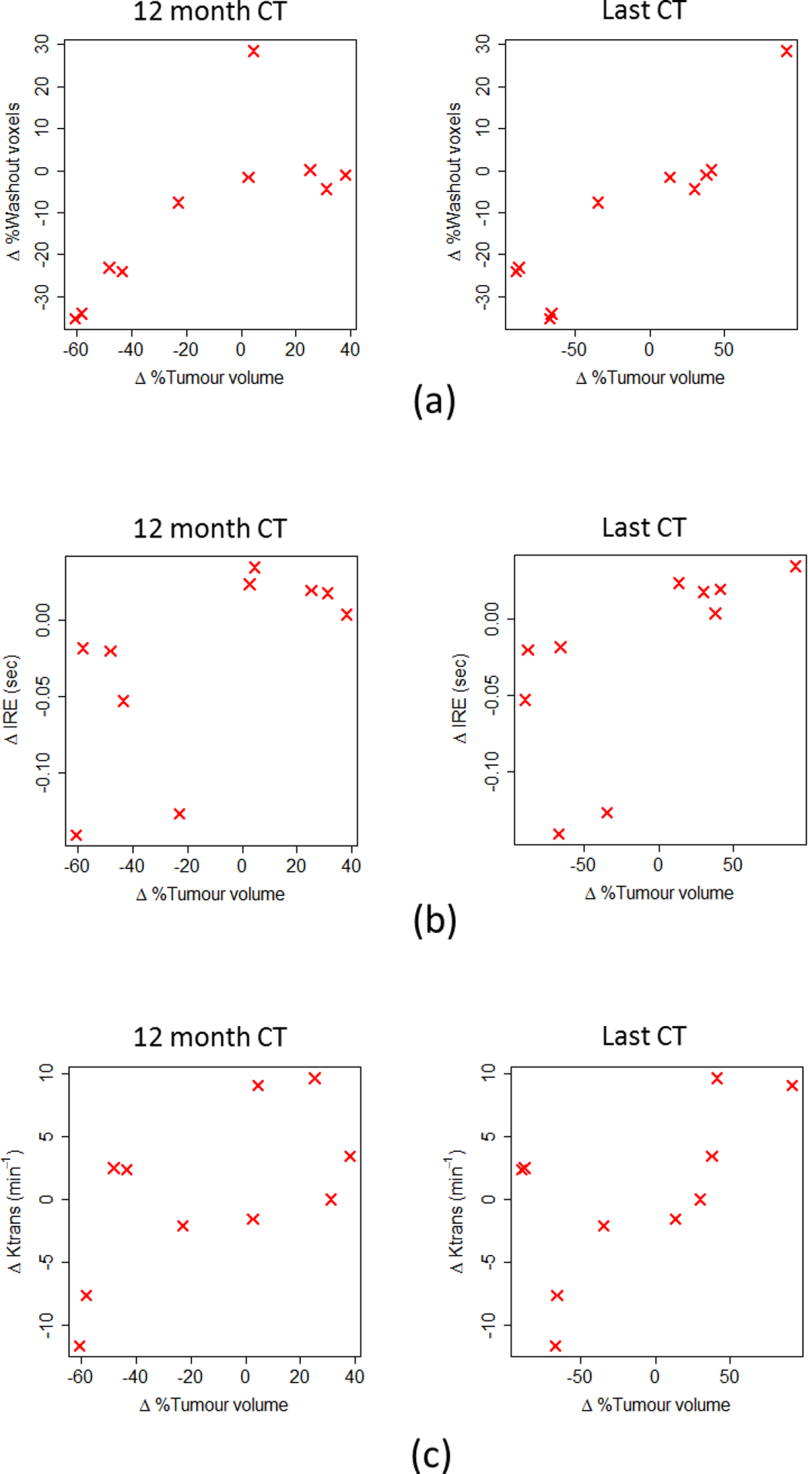
Scatterplots showing the change in (a) percentage washout voxels, (b) mean IRE and (c) mean Ktrans from follow-up MRI scan 2 versus (left–right) percentage tumour volume change at the 12 month and last follow-up CT.

A significant positive correlation was found at the second follow-up MRI scan between the change in percentage tumour voxels having washout contrast enhancement behaviour and the change in tumour volume at the 12 month CT onwards (ρ = 0.84, p = 0.004 at 12 month CT, ρ = 0.81, p = 0.02 at 24 month CT, and ρ = 0.89, p = 0.001 at the last follow-up CT). Fig 5B shows scatterplots for the 12 month CT and last follow-up CT scan data. There was also a consistently positive but weaker correlation between the changes in these variables at the first follow-up MRI scan (ρ = 0.50, p = 0.12 at 12 month CT, ρ = 0.62, p = 0.11 at 24 month CT, and ρ = 0.60, p = 0.07 at the last follow-up CT). Conversely, there was a negative correlation between the change in percentage plateau voxels and the change in tumour volume at the first follow-up MRI scan relative to the last follow-up CT (ρ = -0.75, p = 0.02) which was also negative but weaker at the second follow-up MRI scan (ρ = -0.28, p = 0.43).

**Fig 6:**
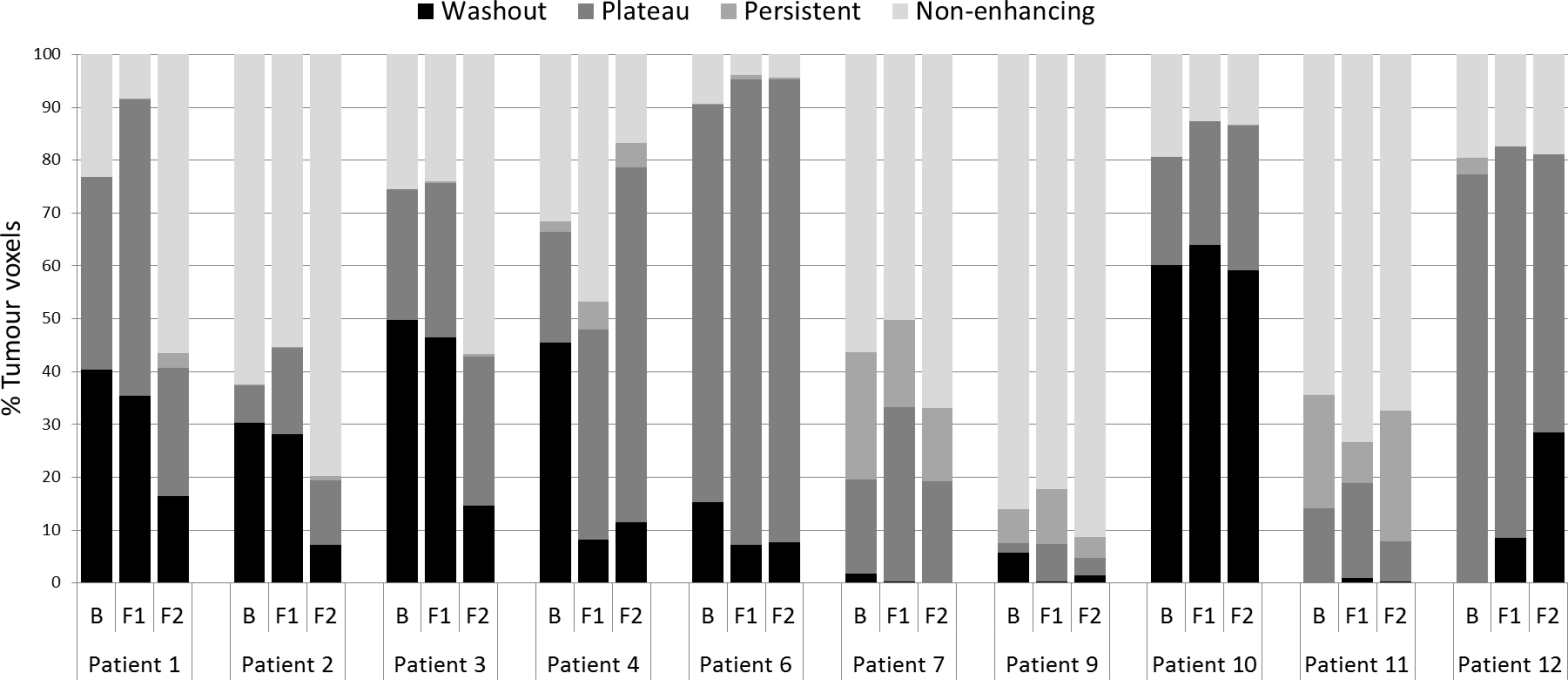
The percentage tumour voxels with each of the four characteristic contrast enhancement curve patterns. B = baseline, F1 = follow-up MRI scan 1, F2 = follow-up MRI scan 2.

Graphs in Fig 6 show the percentage voxels within each tumour ROI which have each of the four contrast enhancement behaviours. All patients with tumour volume shrinkage at last follow-up CT (patients 1 to 6, excluding patient 5 with papillary RCC histology) had a decrease in the percentage of washout voxels at the first and second follow-up MRI scans relative to baseline. A large number of voxels across the patient cohort had non-enhancing voxels (mean 38% for baseline MRI, mean 36% for the first follow-up MRI scan and mean 47% for the second follow-up MRI scan).

The change in mean IRE at the second follow-up MRI scan was positively correlated with change in tumour volume from the 12 month CT scan onwards (ρ = 0.65, p = 0.05 at the 12 month CT, ρ = 0.71, p = 0.06 at the 24 month CT and ρ = 0.78, p = 0.01 at the last follow-up CT) as shown in Fig 5B. In addition, there was a consistent positive correlation between the change in mean Ktrans and the change in tumour volume at all CT time points for the second follow-up MRI scan (Table 3), with the 12 month CT time point being significant (ρ = 0.66, p = 0.04, see Fig 5C). No other correlations were considered statistically significant, and scatterplots for the remaining data can be seen in S3–S6 Figs.

## Discussion

Standard response assessments using morphological measurements and CT based contrast enhancement criteria after SABR for primary RCC are known to be challenging. In a recent study, it was shown that tumour growth rate and tumour size were reduced in primary RCC after SABR, however no significant differences in tumour signal enhancement were shown on multiphasic contrast-enhanced MRI during the initial post treatment period [28]. The authors concluded further studies were needed to determine the correlation between tumour enhancement and disease progression and specified the need for longer follow-up times. Additionally primary RCC may have ongoing tumour regression for many months and years after SABR, rendering early response assessment using conventional RECIST criteria inadequate. Thus there is a clinical need for a more effective biomarker of treatment response than conventional CT imaging after SABR for primary RCC [29]. Such a biomarker could help identify patients who may benefit from adjuvant treatment such as systemic therapy.

Acquiring DWI and DCE MRI of the kidneys to assess their potential as biomarkers for primary RCC, however, poses a number of challenges [14,15]. Currently there are no standardised acquisition protocols for either DWI or DCE MRI, which makes choosing the optimal sequences and assessing prior studies difficult. Respiratory motion is a particularly difficult technical challenge, as acquisition times last for a few minutes which renders the use of breath-hold methods to reduce motion impractical. The cost and availability of MR imaging is a restricting factor in many settings, and it is difficult to obtain large patient cohorts for effective biomarker validation. DCE MRI has extra challenges including an inherent trade-off between the spatial and temporal resolution, and the lack of standardised DCE MRI analysis methods for primary RCC. In addition the uptake of contrast agent in diseased kidneys is typically reduced compared with normal kidneys, causing a decrease in the signal-to-noise ratio in DCE MRI data [30].

Within this challenging context, we sought to carry out an exploratory analysis of diffusion and perfusion changes shown by DWI and DCE MRI after SABR for primary RCC and assess whether any correlate with treatment response shown on CT. We have carried out this assessment on patients with a consistent imaging protocol, acquired on the same MR scanner which has eliminated sequence and scanner variability. We have correlated changes in MRI parameters with percentage tumour volume change from consistent CT time points, while also assessing the last follow-up CT scan which we considered suitable as tumour shrinkage over time is typically slow and relatively linear [28]. This last follow-up CT was a median 761 days (range 385–1085 days) after SABR treatment, providing a long follow-up time for the analysis (see S1 Table).

Radiation is known to induce inflammation in treated tissues, and is a known confounder when assessing response in patients who have undergone SABR for lung cancer [31]. To our knowledge, no studies have investigated how long inflammation lasts in the kidney after SABR for RCC, however we anticipated the first follow-up MRI scan which was acquired at a median 15 days post therapy (S1 Table), was likely to be within the inflammatory window. In contrast the tumour tissue at second follow-up MRI scan, acquired at a median 70 days post therapy (S1 Table), will have had more time to resolve any inflammation. This may explain why correlations for the first and second follow up scans for many of the DWI and DCE parameters were frequently dissimilar and provides a rationale for assessing tumour response once inflammation is likely to have resolved. We therefore hypothesise that follow up imaging may not be informative until the second follow-up MRI scan time which was a median 70 days post SABR, which should be taken into consideration when designing future studies.

Our patient cohort showed restricted diffusion in their tumours on baseline ADC maps from DWI, which is consistent with a meta-analysis for focal renal lesions where ADC values were significantly lower than in benign tissue [32]. Recent studies have also shown that ADC negatively correlates with cell density in many solid malignancies, primarily due to an increased tumour cell proliferation [33]. Therefore we hypothesise the increase in mean and median ADC in many patients after SABR shows a tendency for cell density within tumours to decrease after treatment. Mean or median ADC did not consistently correlate with the change in tumour volume, however, so it did not demonstrate potential as a treatment response biomarker. In contrast the change in ADC kurtosis held the most promise, showing a positive correlation with the change in tumour volume from the 12 month CT time point onwards at both follow up MRI scans with the correlations between the 12 month and last follow-up CT for the first follow-up MRI scan showing significance. However, due to the likely confounding factor caused by inflammation, it is difficult to draw strong hypotheses from these findings.

It is important to consider that ADC was computed using a simplistic mono-exponential decay curve to the DWI data, which assumes diffusion obeys a Gaussian distribution. We know that DWI provides complex mixed information about cellularity and perfusion where low b-values, specifically those less than 200 s/mm^2^, are highly influenced by perfusion effects [34]. To extract more information from DWI imaging to more precisely characterise the microstructure within tumours, non-Gaussian models such a bi-exponential intravoxel incoherent motion (IVIM) model, stretch exponential or a kurtosis model [35] could be used in future. An additional consideration in this patient cohort is pre-existing kidney disease that might lead to risk of contrast nephropathy. In this instance non-contrast models such as IVIM diffusion MR imaging which allow the effects of diffusion and perfusion effects to be separated and assessed would be particularly useful. [35].

High perfusion is a characteristic of tumours due to their abnormal microvasculature, which is reflected by higher signal intensities on DCE MRI. Correlation analysis showed a strong association at the second follow up scan between the change in percentage voxels showing washout contrast enhancement behaviour, which is typical of tumours, and the change in tumour volume at the 12 month CT time point onwards. Furthermore the change in IRE and in Ktrans, which are both dependent on local tissue permeability and perfusion, at this second scan were positively correlated with the percentage change in tumour volume from the 12 month CT scan onwards. With these findings, we hypothesise that patients who experienced tumour growth despite having SABR, still had viable tumour vasculature with angiogenic activity after treatment while patients whose tumours reduced in size likely had successful ablation of the associated perfusing vessels. There was a large amount of tumour tissue with no contrast enhancement located at sites of necrosis, and also in regions affected by motion which impeded data fitting. Methods ensured however, that voxels with no contrast enhancement were not included in the computation of mean DCE parameters so as not to skew the results.

Results here are comparable with those shown in a number of oncological studies investigating the use of DCE MRI for treatment response evaluation after radiotherapy. In a study by Huang et al. [36] DCE-integrated MR-PET was used to assess tumour response in 17 patients who underwent SABR for non-small cell lung cancer. Their results showed the percent reduction in mean pharmacokinetic parameter Ktrans and mean kep (the transfer constant from the extracellular space to blood plasma) 6-weeks post SABR were significantly correlated with percent reduction in tumour size shown on CT imaging 3 months after treatment. Regarding treatment for spine metastases, a study by Spratt et al. [37] analysed DCE MRI data pre and post SABR of 12 lesions from 9 patients. They showed perfusion parameters from DCE MRI provided excellent correlation with local control, with Ktrans max being 100% accurate for predicting later failure while in contrast traditional subjective impressions and size criteria alone were shown to be insufficient to judge ultimate disease progression. In another study, Chu et al. [38] examined changes in spinal metastases in 15 patients using DCE MRI before and after external beam radiotherapy. They showed that pharmacokinetic parameter Vp (the fractional volume of plasma in tissue) was a strong predictor of treatment response, however Ktrans was not significantly correlated. More recently, a study by Lis et al. [39] showed in a small subset of six patients that changes in tumour perfusion as indicated by Ktrans and Vp could be seen within 1 hour of radiotherapy showing tumour changes could be seen in DCE MRI almost immediately.

There were limitations to our study, most notably the small number of patients and the large number of parameters assessed. Hence this study is necessarily of an exploratory nature, and we did not adjust for multiple comparisons. Motion of the kidney during DWI and DCE MRI data acquisition caused mainly by respiration provided challenges to the data analysis. The images were obtained during free-breathing which meant the kidney movement occurred predominantly in the coronal plane [40], which was perpendicular to the axial data acquisition. This motion impacted the pre contrast T1 images for some patients, so that we decided to use a consistent T1 value from literature which may have reduced the accuracy of the Ktrans values. Acquiring images in the coronal plane and implementing post-processing techniques including 3D motion correction algorithms will be investigated in future studies to enhance data quality and the accuracy of computed pharmacokinetic parameters.

We assessed MRI tumour volumes using contours from one observer and extracted mean values for a number of MRI parameters which can be heavily influenced by outliers. Whilst we did investigate tumour contouring on the T2-weighted and post contrast MR images where tumour boundaries could be more easily seen, it was challenging to accurately co-register these images and the contour data to the lower resolution ADC maps and DCE MRI which had been more influenced by motion. Instead by contouring the ADC maps and DCE MRI data the radiologist could visually take the effect of motion into account. It will be a subject of future studies to investigate the impact of contour variation, particularly inter-observer variability. Future studies will also be done into assessing response categorisation using imaging data directly which can then be compared with conventional methods including RECIST, similar to the REMAP study which is assessing treatment response to antiangiogenic therapy for metastatic RCC using combined PET/MRI [41].

Lastly, the time between imaging studies and treatment were inconsistent between patients (S1 Table). Baseline MRI was obtained a median 43 days (range 16–62 days) prior to SABR; the first follow up MRI a median 15 days (range 9–59) after treatment and the second follow up MRI a median 70 days (range 56–162) after treatment. Similarly, the CT scan times for the 6, 12 and 24 month follow-up assessments varied (S1 Table). We considered the differences in MRI and CT scan times relative to treatment was not important, however, due to the slow growing nature of primary RCC tumours.

## Conclusions

To our knowledge, we have carried out the first exploratory study into assessing whether DWI and DCE MRI parameters could provide quantitative early functional imaging biomarkers for SABR treatment of primary RCC. Results showed that parameters from DCE MRI had strong potential as early response biomarkers, with the washout contrast enhancement curve behaviour at 70 days post SABR significantly correlating with the percentage change in tumour volume shown on CT at 12 months and onwards. In addition, the IRE and Ktrans parameters from DCE-MRI at 70 days post SABR showed potential utility for predicting treatment response at 12 months and onwards. ADC kurtosis from DWI at 15 days post SABR was significantly correlated with percentage tumour volume change at the 12 month CT time point however it is possible this scan was confounded by the effects of radiation-induced inflammation. Further prospective studies to assess and validate these potential biomarkers using larger patient cohorts are warranted.

## Supporting information

S1 Fig Schematics showing (a) the piecewise linear function fitted to each voxel in the DCE MRI data after normalising the signal intensity to the average baseline signal intensity, from which parameters are extracted including Tonset = time of onset of the contrast agent, IRE = initial rate of enhancement, ME = maximum enhancement, Twashout = time of washout of the contrast agent and IRW = initial rate of washout; and (b) the contrast enhancement behaviour of each voxel is then categorised into either plateau, persistent, washout or no enhancement, to give a Gadolinium (GD) map. Implemented in Dynamika software (Image Analysis Group, London, UK) [20].
(TIF)

S2 Fig (a) Mean ADC and (b) ADC kurtosis for each MRI scan for each patient. ADC kurtosis is dimensionless.
(TIF)

S3 Fig Scatterplots showing the change in (a) mean ADC, (b) median ADC (c) ADC kurtosis and (d) ADC skewness from follow-up MRI scan 1 (left) and follow-up MRI scan 2 (right) versus percentage tumour volume change from CT.
(TIF)

S4 Fig Scatterplots showing the change in (a) ME, (b) IRE and (c) IRW from follow-up MRI scan 1 (left) and follow-up MRI scan 2 (right) versus percentage tumour volume change from CT.
(TIF)

S5 Fig Scatterplots showing the change in (a) Tonset, (b) Twashout, (c) Ktrans and (d) iAUGC60 from follow-up MRI scan 1 (left) and follow-up MRI scan 2 (right) versus percentage tumour volume change from CT.
(TIF)

S6 Fig Scatterplots showing the change in percentage (a) plateau, (b) persistent (c) washout and (d) non-enhancing voxels from follow-up MRI scan 1 (left) and follow-up MRI scan 2 (right) versus percentage tumour volume change from CT.
(TIF)

S1 Table The number of days between each CT and MRI scan and the start of SABR treatment. (PDF)
