# Supplementary figures and images for: Diffusion weighted and dynamic contrast enhanced MRI as an imaging biomarker for stereotactic ablative body radiotherapy (SABR) of primary renal cell carcinoma
Source: PLoS One. 2018 Aug 16;13(8):e0202387. doi: 10.1371/journal.pone.0202387 (PMC6095575; doi:10.1371/journal.pone.0202387)

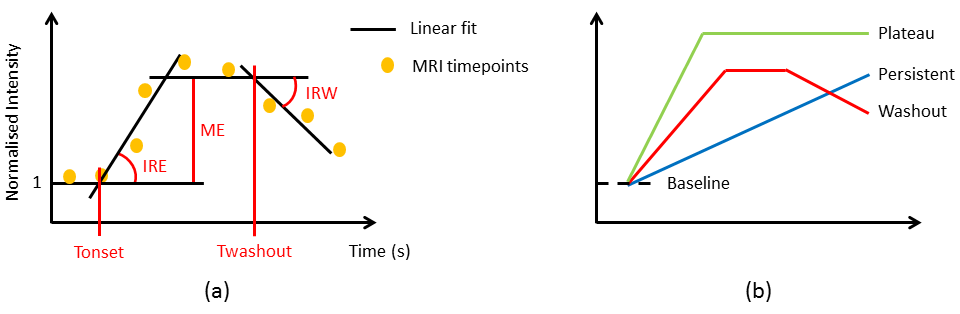

Supplement: S1 Fig — Schematics showing (a) the piecewise linear function fitted to each voxel in the DCE MRI data after normalising the signal intensity to the average baseline signal intensity, from which parameters are extracted including Tonset = time of onset of the contrast agent, IRE = initial rate of enhancement, ME = maximum enhancement, Twashout = time of washout of the contrast agent and IRW = initial rate of washout; and (b) the contrast enhancement behaviour of each voxel is then categorised into either plateau, persistent, washout or no enhancement, to give a Gadolinium (GD) map. Implemented in Dynamika software (Image Analysis Group, London, UK) [20]. (TIF) [file pone.0202387.s001.tif]

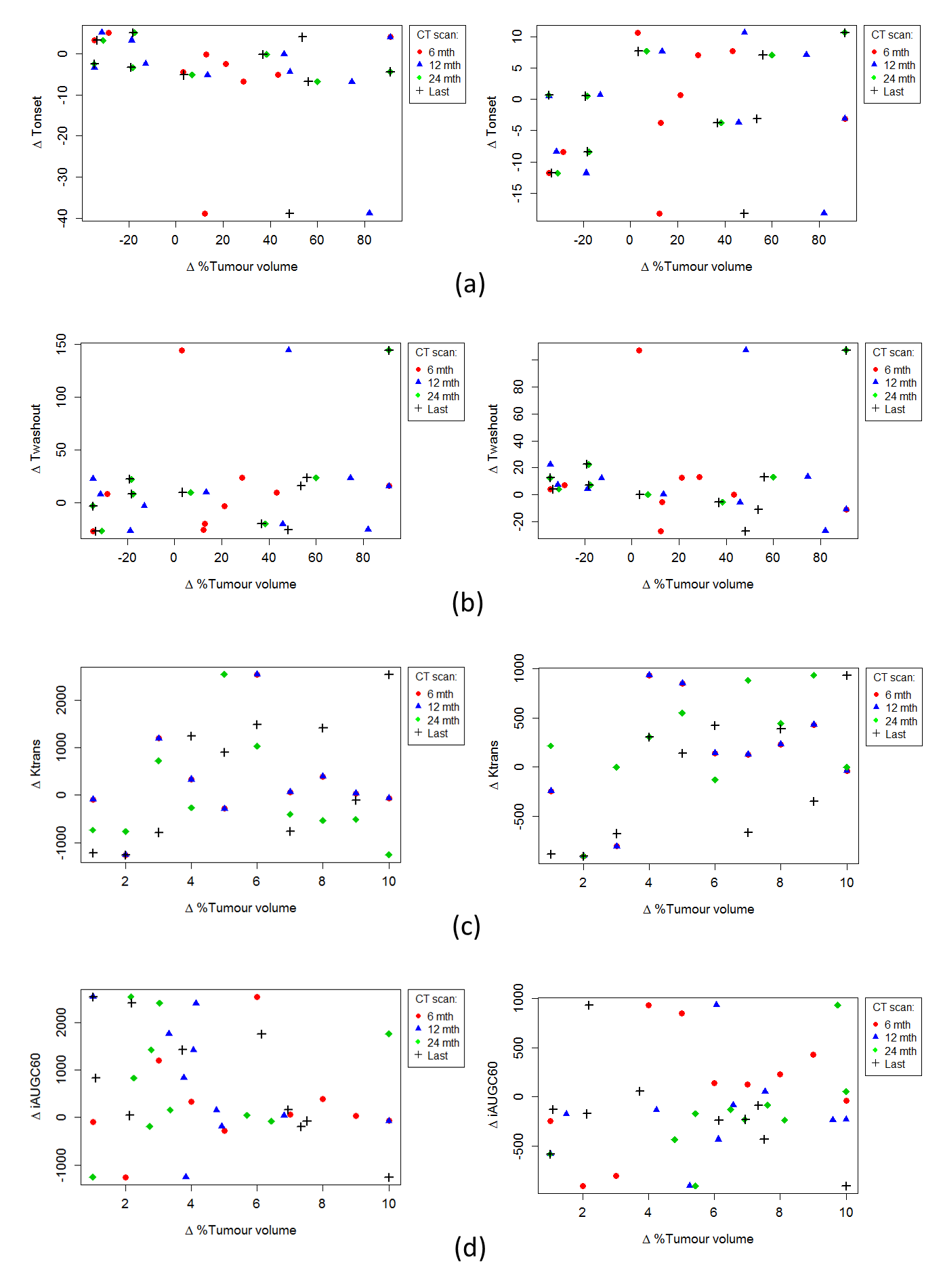

Supplement: S5 Fig — Scatterplots showing the change in (a) Tonset, (b) Twashout, (c) Ktrans and (d) iAUGC60 from follow-up MRI scan 1 (left) and follow-up MRI scan 2 (right) versus percentage tumour volume change from CT. (TIF) [file pone.0202387.s005.tif]

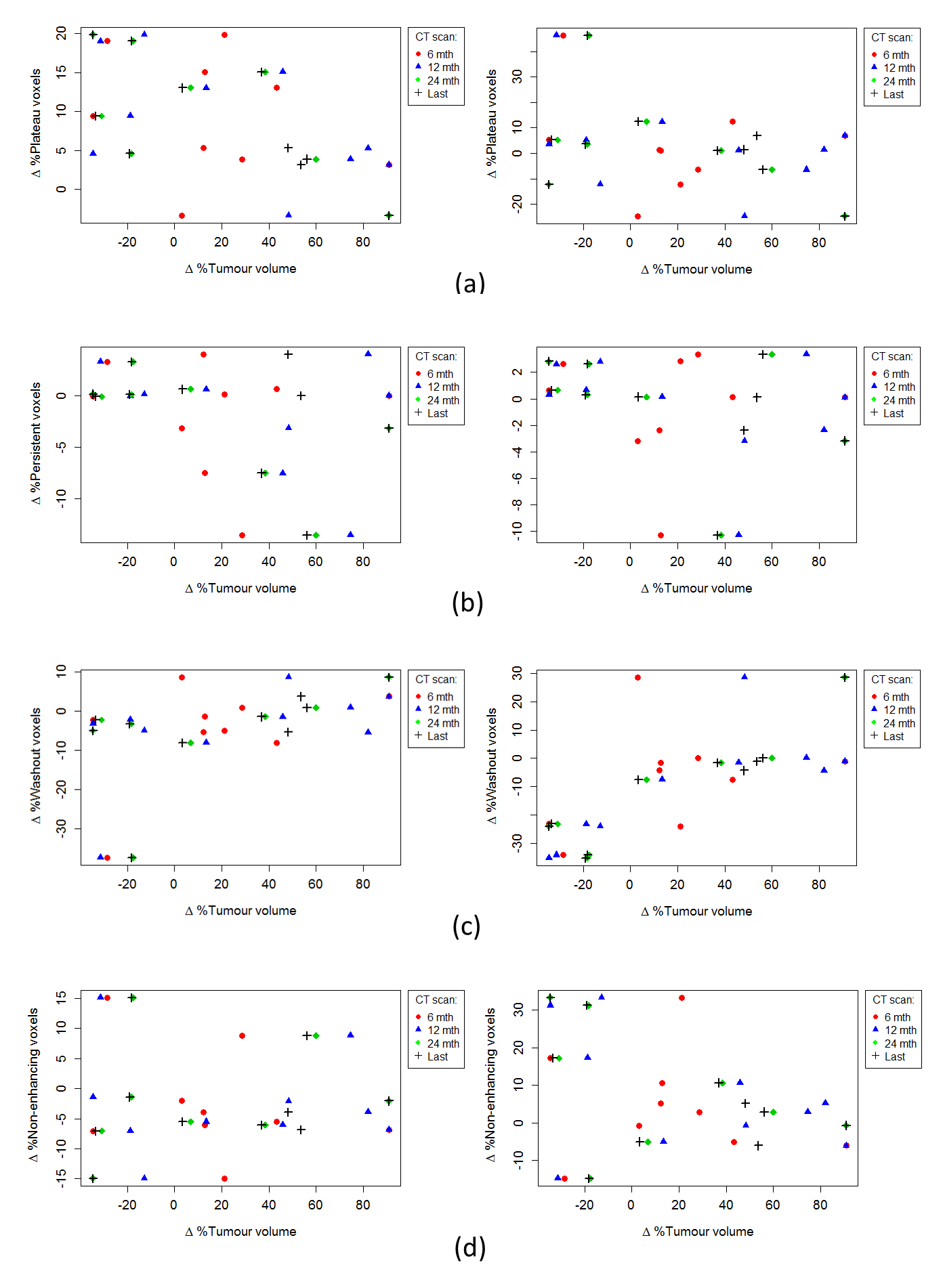

Supplement: S6 Fig — Scatterplots showing the change in percentage (a) plateau, (b) persistent (c) washout and (d) non-enhancing voxels from follow-up MRI scan 1 (left) and follow-up MRI scan 2 (right) versus percentage tumour volume change from CT. (TIF) [file pone.0202387.s006.tif]
