# Supplementary material for: Diffusion weighted and dynamic contrast enhanced MRI as an imaging biomarker for stereotactic ablative body radiotherapy (SABR) of primary renal cell carcinoma
Source: PLoS One. 2018 Aug 16;13(8):e0202387. doi: 10.1371/journal.pone.0202387 (PMC6095575; doi:10.1371/journal.pone.0202387)
Supplement: S1 Table — (PDF) [file pone.0202387.s007.pdf]

Table S1: The number of days between each CT and MRI scan and the start of SABR treatment.

	CT					MRI		
**Patient #**	**Baseline CT (days)**	**6 month CT (days)**	**12 month CT (days)**	**24 month CT (days)**	**Last CT (days)**	**Baseline MRI (days)**	**Follow-up MRI scan 1 (days)**	**Follow-up MRI scan 2 (days)**
1	-79	200	388	794	794	-51	17	69
2	-99	173	364	831	1085	-56	9	70
3	-77	-	367	739	739	-62	14	162
4	-71	144	339	724	724	-52	39	68
5	-90	-	355	694	883	-37	32	112
6	-180	202	377	748	790	-49	14	68
7	-119	212	405	677	782	-28	14	62
8	-134	192	384	733	733	-28	14	119
9	-100	138	355	-	544	-53	16	77
10	-133	182	385	-	385	-29	59	119
11	-56	168	357	715	715	-16	14	56
12	-231	149	364	661	1010	-23	15	70
**Median (min, max)**	**-100 (-56, -231)**	**178 (138, 212)**	**366 (339, 405)**	**729 (661, 831)**	**761 (385, 1085)**	**-43 (-62, -16)**	**15 (9, 59)**	**70 (56, 162)**
